# Generalized longitudinal susceptibility for magnetic monopoles in spin ice

**DOI:** 10.1098/rsta.2011.0596

**Published:** 2012-12-28

**Authors:** Steven T. Bramwell

**Affiliations:** London Centre for Nanotechnology and Department of Physics and Astronomy, University College London, 17–19 Gordon Street, London WC1H 0AJ, UK

**Keywords:** spin ice, magnetic monopoles, magnetic susceptibility

## Abstract

The generalized longitudinal susceptibility *χ*(q,*ω*) affords a sensitive measure of the spatial and temporal correlations of magnetic monopoles in spin ice. Starting with the monopole model, a mean field expression for *χ*(q,*ω*) is derived as well as expressions for the mean square longitudinal field and induction at a point. Monopole motion is shown to be strongly correlated, and both spatial and temporal correlations are controlled by the dimensionless monopole density *x* which defines the ratio of the magnetization relaxation rate and the monopole hop rate. Thermal effects and spin-lattice relaxation are also considered. The derived equations are applicable in the temperature range where the Wien effect for magnetic monopoles is negligible. They are discussed in the context of existing theories of spin ice and the following experimental techniques: DC and AC magnetization, neutron scattering, neutron spin echo and longitudinal and transverse field *μ*SR. The monopole theory is found to unify diverse experimental results, but several discrepancies between theory and experiment are identified. One of these, concerning the neutron scattering line shape, is explained by means of a phenomenological modification to the theory.

## 1. Introduction

Following the paper of Castelnovo, Moessner and Sondhi [1] on emergent magnetic monopoles, there has been renewed interest in the properties of spin ice [2–5]. Magnetic monopole currents were first envisaged by Ryzhkin [6], while Jaubert & Holdsworth [7,8] studied the closely related problem of magnetic relaxation in spin ice by means of numerical simulations of the dipolar spin-ice model [9,10] and of a dual monopole electrolyte. Evidence for the characteristic non-Ohmic conductivity signature of a weak monopole electrolyte—the Wien effect—was reported in [11,12], where the term ‘magnetricity’ was coined.

Experimental evidence indicates that magnetic monopoles afford an economical description of spin ice at temperatures below approximately 10 K [13–15]. In particular, down to about 0.3 K the equilibrium specific heat is well described by the Debye–Hückel theory [14,16,17]. However, to account in detail for the monopole currents and magnetic relaxation is generally a tricky problem, especially in the regime of slow dynamics at sub-kelvin temperatures, and this is an ongoing subject of investigation [12,18,19].

Prior to the recent wave of interest, the spin dynamics of spin ice were studied in detail by Matsuhira *et al.* [20] and Snyder *et al.* [21] by AC susceptibility, by Ehlers *et al.* [22,23] using neutron spin echo, by Lago *et al.* [24] using muon spin relaxation (μSR), by Orendáč *et al.* [25] using bulk magnetocalorimetric methods, by Kitagawa *et al.* [26] using nuclear quadrupole resonance, and by Sutter *et al.* [27] using nuclear forward scattering. Previous studies of the spatial spin correlations by neutron scattering may be found, for example, in [2,[28–33].

The spin correlations in the spin-ice state are characterized by two remarkable features [13,14,34,35]. The first is a property common to many ice-type models—that transverse magnetization (or polarization) fluctuations are essentially unrestricted while longitudinal fluctuations are strongly suppressed at low temperature. This behaviour is captured in the phenomenological theory of Youngblood & Axe [36] (formulated to describe ice rule ferroelectrics), in which the deconfined defects do not carry any Coulombic charge. The second remarkable property, of course, is that in spin ice these defects do carry a magnetic Coulomb charge [1,6]. However, they are also associated with a ‘Dirac string network’ of spin configurations that, while not pairing the monopoles, does restrict their motion to some extent [7,17,37].

Any complete theory of spin ice needs to account for the difference between longitudinal and transverse correlations, the Coulombic interactions of monopoles and the effect of the Dirac string network. However, different experiments may pick out one or another of these three properties, so approximate models are useful. If monopoles are the focus, then it is of most interest to discuss the longitudinal response as this is a highly sensitive measure of the spatial and temporal correlation of magnetic monopoles, as shown below. The simplest approach to treating the monopole correlations is a ‘magnetolyte’ model of freely diffusing magnetic charges, in which the effect of Dirac strings is subsumed into the transport coefficients, and electrolyte properties such as Debye–Hückel screening [17], Bjerrum pairing [11,16,17] and the Wien effect [11,12] may be naturally formulated. Another (and earlier) approach [6] accounts for the ignored spin degrees of freedom in the form of an effective reaction field, and this approach has recently been developed to include magnetic charge screening [38].

The aim of the present work is to calculate a generalized longitudinal susceptibility *χ*(***q***,*ω*) for magnetic monopoles in spin ice and to explore its application to experiment. The theory described here is only a modest extension of the earlier approaches of Ryzhkin [6] and Castelnovo *et al.* [17], but one that is necessary for the purpose of comparing theory with experiment. A useful by-product of this work is a clarification of the relationship between these two approaches, and their relation to that of Youngblood & Axe [36]. The equations discussed here are valid at temperatures that are sufficiently high to avoid the non-equilibrium physics of the Wien effect^1^ for magnetic monopoles (more than 0.4 K for Dy_2_Ti_2_O_7_) [11], but not so high that high-energy relaxation processes become important (more than 10 K for Dy_2_Ti_2_O_7_) [17]. The results of the present paper are applicable to zero and weak applied field (*μ*_0_*H*≪1 T).^2^

## 2. Ryzhkin's approximation

Ryzhkin [6] explored the monopole dynamics of spin ice by applying the Jaccard theory of water ice defects in the water ice–spin ice analogy. He showed that the magnetic current density ***J***=***J***_+_+***J***_−_ is related to the rate of change of magnetization ***M*** by the equation (in our notation)
2.1
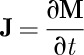
and he derived the rate of entropy production associated with the flow of the magnetic charges
2.2
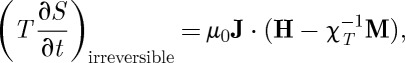
where *S* is entropy and ***H*** is magnetic field. He finally used these relations to derive a thermodynamic equation of motion
2.3
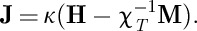
Here *κ*=*ucQμ*_0_ is the monopole conductivity, *u*=*u*_+_=−*u*_−_ is the monopole mobility, *c*=*c*_+_+*c*_−_ is the total concentration of free monopoles and *Q*=*Q*_+_=−*Q*_−_ is the monopole charge. The isothermal susceptibility is predicted to be *χ*_*T*_=2*χ*_C_ where *χ*_*C*_ is the nominal Curie susceptibility for the spin-ice system (*χ*_C_≈3.95/*T* for Dy_2_Ti_2_O_7_).

Equation (2.3) contains much physics and deserves subtle appreciation. The term in ***H*** represents the normal drift current of the charges *Q*_±_ in the applied field ***H***, and if there were only this term, then spin ice would be represented as a true conductor, precisely equivalent to an electrolyte. However, the term in ***M*** opposes the direct current and indeed extinguishes it completely at equilibrium, where *M*=*χ*_*T*_***H***. This reaction field does not originate in the magnetic monopoles themselves but rather in the configurational entropy of the monopole vacuum: magnetization of the system reduces that entropy and hence provides a thermodynamic force that opposes the current [6]. It should also be noted that what stops the current in Ryzhkin's formulation is not the sample boundaries: this is correct under the approximation that the system is homogeneous and linear. If one further allows the competition of diffusion and drift to set up charge density gradients, then the boundaries immediately become relevant and one must additionally consider boundary conditions that do not allow the passage of monopoles. However, this is not necessary in the approximation considered. Finally, it should be emphasized that the extinction of the current implied by equation (2.3) holds only for very small field and magnetization, for it is only in this limit that equation (2.2) is valid.

At first sight, the right-hand side of equation (2.3) is zero but this is only true at infinite time. By introducing a frequency Fourier transform of the magnetization and field and combining equation (2.1) with equation (2.3), Ryzhkin found that
2.4
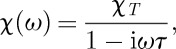
where the inverse relaxation time *τ*^−1^=*κ*/*χ*_*T*_. Equation (2.3) can also be integrated to predict the magnetic response to the sudden application or removal of a uniform field (see §12). Assuming an ellipsoidal sample, when a uniform external field ***H***_ext_ is applied, the internal field ***H***_int_ is reduced by the demagnetizing field 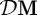, such that 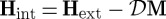. In spin ice the demagnetizing field arises from the magnetic pole density associated with uncompensated surface monopoles. As the spin-ice sample is magnetized, an imbalance of magnetic charge develops at opposite surfaces as a result of the transient monopole current described in equation (2.3). However, as a result of the entropic ‘reaction field’ discussed above, the monopoles are not sufficiently free to achieve complete screening of the internal field. The incomplete screening of the internal field owing to magnetic monopoles has been discussed in detail by Ryzhkin & Ryzhkin [38].

## 3. Definition of the two characteristic rates *ν* and *ν*_0_

We may define the relaxation rate *ν* by
3.1
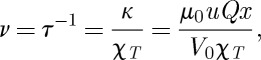
where the concentration *c* has been substituted for the total dimensionless monopole density or mole fraction *x*=*cV*_0_, and *V*_0_ is the volume per site of the diamond lattice inhabited by the magnetic monopoles: 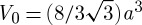, where *a* is the near-neighbour spacing on the diamond lattice.

Using the Nernst–Einstein relation, the mobility *u* may be replaced by the diffusivity *D*:^3^
3.2
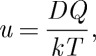
and hence
3.3
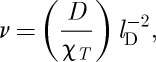
where *l*_D_ is the Debye length:^4^
3.4
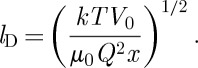


In turn *D* is determined by the monopole hopping frequency *ν*_0_. In a simple lattice diffusion approximation, we may write [12]
3.5
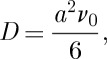
where *a* is the diamond lattice constant (the numerical factor of 6 may be modified very slightly when the fact that a monopole may only hop in three out of four local directions is accounted for [17]). Using the definitions *Q*=2*μ*/*a* where *μ* is the rare-earth magnetic moment, and *χ*_C_=*μ*_0_*μ*^2^/3*V*_0_*kT*, equations (3.1), (3.2) and (3.5) may be rearranged to give
3.6
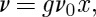
where 
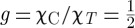
 in Ryzhkin's theory but is more generally weakly temperature dependent and varies between *g*=1 at high temperature and *g*=2 at low temperature [43]. Equation (3.6) will be seen to be very important for the interpretation of experiments on spin ice.

## 4. Coulombic correlation of the monopole current

We define the flux of positive and negative monopoles as ***j***_+_ and ***j***_−_, respectively. Assuming that there is no temperature gradient in the system, then the thermodynamic equations of motion are (with *i*,*j*=+,−)
4.1
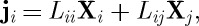
where ***X*** denotes a generalized thermodynamic force. If we assume that the monopole density is small, then, following the theory of weak electrolytes, we would expect the cross terms *L*_*ij*_ with *i*≠*j* to be zero. However, the monopole motion should be strongly correlated in the sense that it always acts to maintain local charge neutrality:
4.2
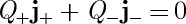
and
4.3
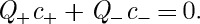
It is important to emphasize that this thermodynamic force is not the same as Ryzhkin's reaction field m which is a purely spin phenomenon peculiar to spin ice. In fact, equation (2.3) does not account for Coulombic correlation between magnetic monopoles and in the next level of description this needs to be accounted for.

The left-hand side of equation (4.2) is simply the magnetic diffusion current ***J***_diffusion_ which contributes to the total magnetic current ***J***=∂***M***/∂*t*. At equilibrium in zero applied magnetic field, the monopole diffusion is such that it does not change the local magnetization of the system. Thus positive and negative monopoles tend to move in the same direction. After a perturbation, the local magnetic current relaxes to zero, even though the monopoles continue to hop around the system. The magnetic diffusion current is given by
4.4
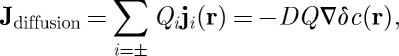
where *δc*(***r***)=*c*_+_(***r***)−*c*_−_(***r***). In the zero field equilibrium state the average local gradient of charge density is everywhere zero.

## 5. Spatial dependence of longitudinal magnetization

The Coulombic correlations create a diffusion force that tends to smooth the local longitudinal magnetization. This may be seen as follows. By the Helmholtz theorem the vector field ***M***(***r***) can be decomposed into an irrotational or longitudinal (i) part and a solenoidal or transverse (s) part,
5.1
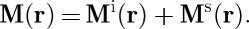
The spin-ice ground state is defined by the condition *M*^i^(***r***)=0, which gives
5.2
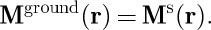
Physically, this is a consequence of the spin-ice ground state consisting of closed loops of spins. The irrotational or longitudinal part is finite only as a result of thermal excitation of magnetic monopoles. As ∇×*M*^i^(***r***)=0, the vector Laplacian is simply
5.3
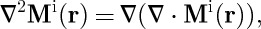
a result that will be used below. Henceforth (unless otherwise stated), we shall only deal with the longitudinal magnetization and the superscript i will be dropped.

Defining *ϕ*(***r***) as the magnetic scalar potential, the local magnetic field is
5.4
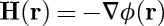
and, by Poisson's equation and Maxwell's equation, the local magnetic charge density is
5.5
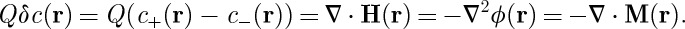
Thus, using equation (5.3), the local charge density gradient is
5.6
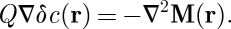
The magnetic diffusion current associated with a finite charge density gradient is
5.7
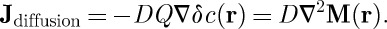
This term can then be added to equation (2.3) to describe relaxation of the spatial charge arrangements,
5.8
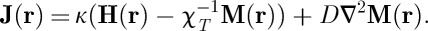
In recent work, Ryzhkin & Ryzhkin [38] stated such an equation to facilitate a calculation of magnetic screening effects in spin ice.

## 6. Free energy functional

The same equation can be derived from a Landau-type free energy functional as follows. If we apply a local field ***H***(***r***), then this induces a longitudinal response ***M***(***r***). The local magnetization is opposed by the entropy cost of ordering the spins of the sample as well as the entropy cost of creating a local charge imbalance. Note that these two factors are distinct: it is possible to increase order in the sample without creating a local charge imbalance.

From general chemical thermodynamics, we expect the entropic cost of charge imbalance to make the following contribution to the local Gibbs free energy:
6.1
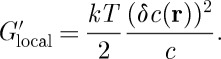
Hence, using Poisson's equation and Maxwell's equation again,
6.2
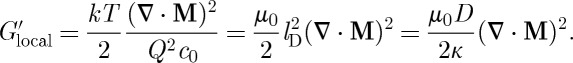
We may then write down a free energy functional for the system,
6.3
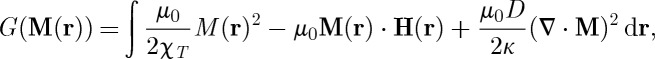
where the first term on the right-hand side represents the Jaccard entropy [6] which in this representation is seen to be equal to the entropy of a cooperative paramagnet [44]. The rate of change of longitudinal magnetization ***M***(***r***) may be found in a linear response approximation,
6.4
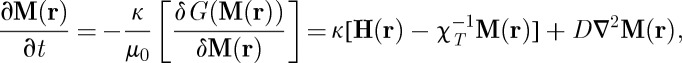
which gives equation (5.8). The derivation of the right-hand term of equation (6.11) is given below,^5^ from which it can be seen that the contribution of surface charge to the Gibbs energy is neglected.

Under conditions of fixed temperature and field, the rate of dissipation is
6.10
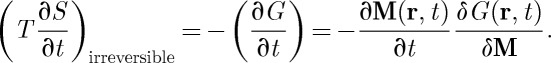
Hence, using equation (2.1),
6.11
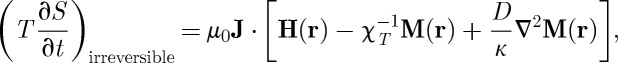
which is the extension of equation (2.2) to include monopole diffusion.

Owing to the neglect of surface charge, these equations are generally applicable only under conditions of small field and small magnetization, or else at short time. When these conditions are violated, the build up of surface charge may have a decisive influence on the internal fields and on the magnetization process, and the preceding equations would need to be modified to account for this.

## 7. Generalized longitudinal susceptibility

We introduce the spatial and time-dependent Fourier transforms of the longitudinal magnetization and longitudinal magnetic field,
7.1
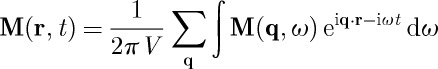
and
7.2
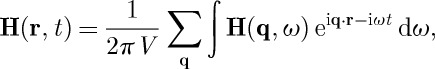
and the generalized susceptibility (assuming translational invariance),
7.3
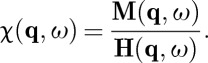
Substituting these definitions into equations (5.8) and (2.1), we find
7.4
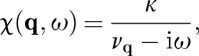
where
7.5
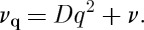
Note that a generalized susceptibility of this sort could also be derived by solving a Langevin equation incorporating the Landau free energy, as described by Goldenfeld [45].

Using equation (3.6), the generalized susceptibility can also be written
7.6
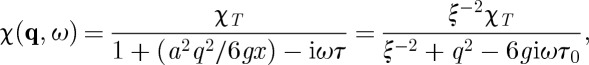
where *τ*_0_=1/*ν*_0_ and the correlation length is
7.7
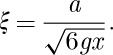


It should be emphasized that this is an equation for the longitudinal susceptibility only (***M***∥***H***∥***q***). Whether at equilibrium or not, the latter is finite only if there is a finite density of monopoles. By contrast (see §5), the transverse susceptibility of spin ice could take a paramagnetic value at equilibrium even in the complete absence of monopoles, as it does in the monopole-free spin-ice ground state. In principle, the transverse susceptibility could relax through the flipping of closed loops of spins [3], though in reality it is more probable that it relaxes through the transient passage of magnetic monopoles. A more complete description of the wavevector dependence of the susceptibility is given in [13,36,44,46], and the possibility of quantum mechanical effects giving rise to transverse dynamics distinct from magnetic monopoles (so called ‘photons’) has been discussed in [47,48].

There is potentially a problem with equation (7.5) and the subsequent equations. To see this, we rewrite *ν*_***q***_ as follows:
7.8
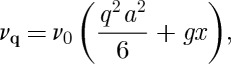
and assume that the maximum possible equilibrium value of the density is 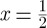. Since it seems implausible that *ν*_***q***_ would ever exceed *ν*_0_ (and indeed *ν*_***q***_ should generally be less than it), then it appears that equation (7.5) breaks down at *q*^2^*a*^2^>3 *g*. To guarantee that this never happens we can write
7.9
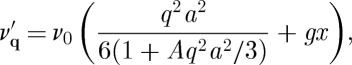
where *A* is introduced as a phenomenological (dimensionless) parameter. Applying equation (7.4) we find
7.10
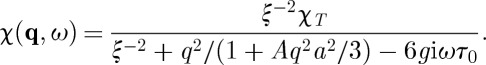
As discussed further below (§14), it would seem more realistic to use this phenomenological equation than equation (7.6) in order to describe the experiment.

## 8. Equilibrium field fluctuations owing to monopoles

Consider now the spin-ice state in zero applied magnetic field, where the internal field ***H***(***r***), which originates from the magnetic monopoles, is no longer constrained but instead relaxes self-consistently with the magnetization. An approximation to the problem is that, close to equilibrium, the field takes the value 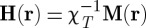 everywhere, and that this relation is maintained for small fluctuations away from equilibrium. The internal field therefore costs spin entropy
8.1
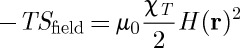
and energy
8.2
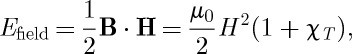
but this is offset by the energy gain in magnetizing the sample,
8.3
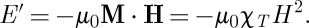
Summing these contributions and allowing the magnetic charge density to fluctuate along with the field, we find a free energy functional for field fluctuations,
8.4
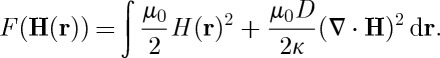
This functional is entirely equivalent to that for electric field fluctuations in an electrolyte and in fact is equivalent to Debye–Hückel theory [49]. Thus, we see that by suppressing spin fluctuations (i.e. setting 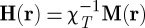) we recover the Debye–Hückel approximation of Castelnovo *et al.* [17].

Introducing the Fourier transformed field 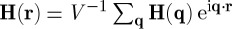 and substituting into equation (8.4), we find
8.5
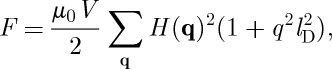
where *V* is the volume and we have used 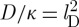 and the fact that the field, being derived from a scalar potential, is longitudinal to the wavevector. Since the probability of a fluctuation is ∝*e*^−*F*/*kT*^, we immediately see that the mean square amplitude of a mode is
8.6
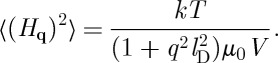
The mean square field at a point is
8.7
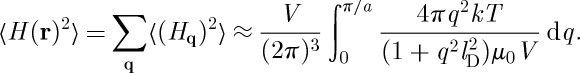
The integral is dominated by short wavelength modes and the mean square field approximately takes the value
8.8
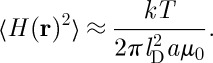


## 9. Mean square induction at a point

Using equation (8.8), the mean square induction at a point is
9.1
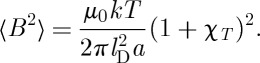
Obviously, this equation depends on the induction being averaged over a sufficiently large volume that contains locally magnetized spins. If the point with which we are concerned experiences no local induction from the magnetized spins, and sees only a far field, then the mean square induction is simply
9.2
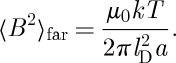
Recalling that 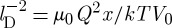 where *V*_0_∼*a*^3^ it may be shown that
9.3
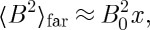
where
9.4
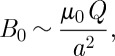
and the symbol ∼ is used to indicate that factors of order unity are dropped. In the case that there is local induction arising from magnetized spins, this equation may be modified to
9.5
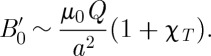


It is instructive to derive equation (9.3) in direct space. At a point in the sample the squared field may be averaged over contributions at distance *r* weighted by the probability of finding a monopole at that distance:
9.6
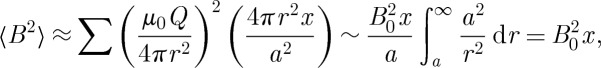
which neglects correlation between the field contributions. The fields are correlated over a distance of *l*_D_, but the average number of monopoles within a distance *l*_D_ is typically of order unity, so correlation may be neglected to a first approximation. The field *B*_0_ is approximately that owing to a monopole or a spin at a distance *a*. Thus, if a defect is viewed at a distance *a* it looks like a spin, but if viewed at a much greater distance it looks like a monopole. For this reason, monopoles are best detected by measuring their far fields [11].

## 10. Relaxation of the field fluctuations

The equivalent electrolyte theory has been formulated and worked out in detail by Oosawa [49], who found that the relaxation rate of a mode labelled by ***q*** is
10.1
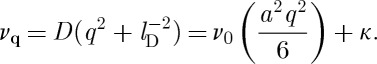
Thus, short wavelength modes relax at a rate of *ν*_0_, the monopole hop rate, and long wavelength modes relax at a rate *κ*, the monopole conductivity. Fluctuations are important on all scales between the lattice constant and the Debye length, so there is a dispersion of relaxation rates from the monopole hop rate *ν*_0_ to the bulk field relaxation rate or monopole conductivity *κ*.

From equation (8.7), it may be seen that monopoles at distance *a* and those at distance *l*_D_ make similar contributions to the mean square field, while those at much greater distance may be neglected (in zero applied field). Monopoles at distance *a* reverse the local field at a rate of approximately *ν*_0_, whereas the cloud of monopoles at distance *l*_D_ only reverses the field at the much slower rate *κ*, although it gives rise to small field fluctuations at a rate *ν*_0_.

Comparison of equation (10.1) with equation (3.1) suggests that the field correlations relax at a rate *κ* while the spin correlations relax at a rate *ν*=*κ*/*χ*_*T*_. Although this difference may reflect the approximations made, it also seems plausible that the spin correlations relax more slowly than the field correlations at low temperature. Thus, the spin system can only relax by the passage of monopoles, so the time taken to find the most probable spin arrangement will generally be longer than the time taken to find the most probable monopole arrangement.

## 11. Spin-lattice relaxation

It is obvious that the relaxation rate *ν* is equal to the spin-lattice relaxation rate 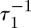 but it is useful to see how this arises in detail. Initial application of a magnetic field *H* should result in almost instantaneous magnetization, in which energy is stored within the system of effective spins. The spin temperature *T*_s_ is therefore initially higher than the applied bath temperature *T*. Following Casimir and du Pré [50] and Morrish [51], the temperature difference *θ*=*T*_s_−*T* determines the rate of exchange of heat 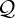 with the thermal bath, and the consequent return of the spin system to thermal equilibrium at temperature *T*,
11.1
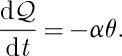
We may also write
11.2
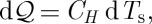
where *C*_*H*_ is the heat capacity at constant field. Hence, we find
11.3
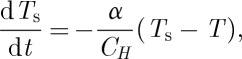
and the spin temperature relaxes at a rate 
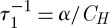
.

To link this to the magnetization relaxation, we use the thermodynamic relations
11.4
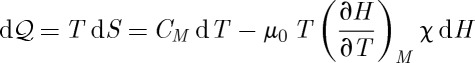
and
11.5
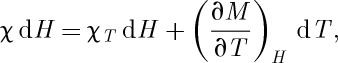
where *χ* d*H*=d*M* and *C*_*M*_ is the heat capacity at constant magnetization. If then we apply a field *H*=*H*_1_ e^i*ωt*^ and elicit a response *θ*=*θ*_1_ e^i*ωt*^ the above two equations may be solved with the substitutions 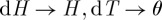, 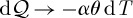 (equation (11.1)) and use of the thermodynamic relation *C*_*H*_−*C*_*M*_=−*μ*_0_ *T*(∂*H*/∂*T*)_*M*_(∂*M*/∂*T*)_*H*_. This gives the well-known result [51]
11.6
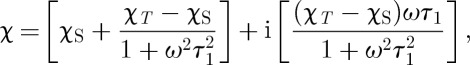
where *χ*_S_=*χ*_*T*_*C*_*M*_/*C*_*H*_ is the adiabatic susceptibility. By comparison with equation (2.4), we see that in Ryzhkin's approximation 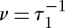 and the adiabatic susceptibility is assumed to be zero. The former is easily understood as any magnetization involves the passage of a monopole current accompanied by dissipation. However, it is conceivable that the adiabatic response could be finite in the real material, and involve the ‘stretching’ of the excited state magnetic moment along the field direction, in which case the adiabatic susceptibility *χ*_S_ could be a direct measure of the density of excited states or monopoles. This idea needs to be checked in detail.

### 11.1 Phonon bottleneck

We may also modify this approach to include a ‘phonon bottleneck’. The spin system is considered to be connected to the phonon system at temperature *T*_p_, and the phonon system is connected to the bath at temperature *T*. The thermal relaxation between the phonon system and the bath is characterized by a thermal conductivity *α*′. If we make a steady-state approximation to the phonon temperature, then the rate of heat exchange between the phonon system and the bath is simply
11.7
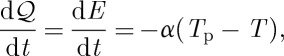
where *E*=*U*−*μ*_0_*MH* is the magnetic enthalpy. Under the approximation that the monopole internal energy *U* is constant, we simply find that
11.8
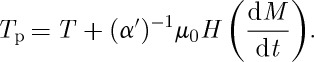
Thus, a thermometer placed on the sample could be used to measure *T*_p_ and hence gain an alternative measure of the magnetic current d*M*/d*t* after transients have died away.

The rise in temperature of the sample (equation (11.8)) occurs when the rate of flow of heat between the spin and phonon systems exceeds the rate of flow of heat from the phonon system to the bath. In the steady-state approximation, the criterion for this is *α*′<*α*=*νC*_*H*_. In the low-temperature limit, we find *C*_*H*_≈(|*μ*|/*kT*^2^*V*_0_)*x* [16], where *μ* is the monopole chemical potential [1,7] and hence
11.9
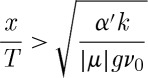
is a criterion for observation of this effect. The ratio *x*(*T*,*H*=0)/*T* is always sufficiently small that this analysis suggests that the bottleneck can never be observed in zero applied field, and hence any observation of a bottleneck is likely to reflect a significant field-induced increase in *x*(*T*,*H*) (the Wien effect). This conclusion is consistent with the experimental observations of Slobinsky *et al.* [52], who observed a phonon bottleneck, albeit in fields much stronger than those appropriate to the theory discussed here.

### 11.2 Thermal quench

If bound monopole pairs equilibrate sufficiently quickly with the monopole vacuum, then the magnetic monopoles may be regarded as in direct equilibrium with the vacuum: (0)=(+)+(−). The equilibrium constant is
11.10
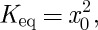
where we temporarily label the equilibrium density as *x*_0_. By definition, the thermodynamic equilibrium constant is given by *K*_eq_=e^2*μ*/*T*^, where *μ*<0 is the monopole chemical potential [1,7].

**Figure 1.**
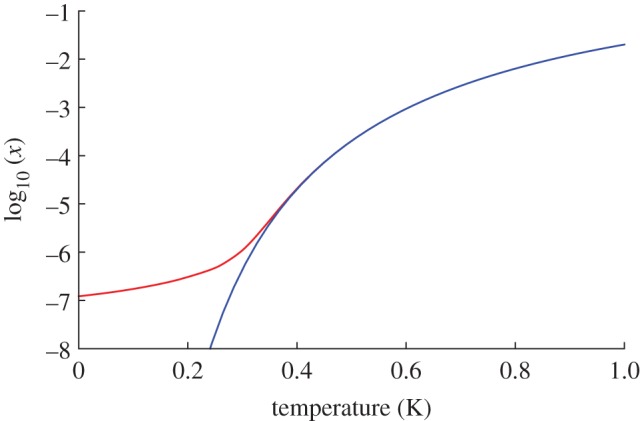
Thermal evolution of the monopole density *x* according to equation (11.12) (red line) versus the equilibrium density 
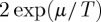
 (blue line), with the cooling process starting at 1.0 K at a rate 10^−5^ K s^−1^ (here *ν*_0_=10^3^ s^−1^, *μ*=−4.6 K).

**Figure 2.**
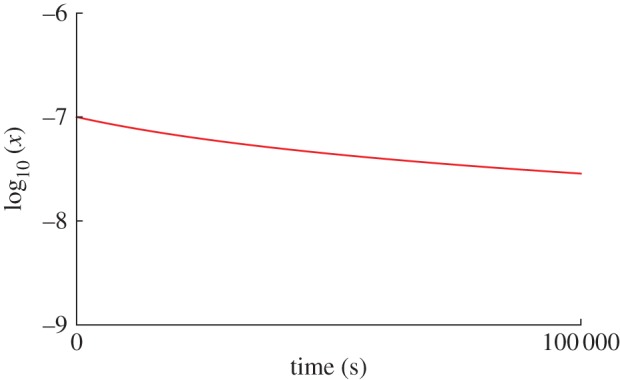
Temporal evolution of the monopole density *x* starting at *x*=10^−7^, according to equation (11.13) (parameters as in figure 1).

Neglecting Bjerrum pairs [12], the kinetic rate equation for the change in monopole density is
11.11
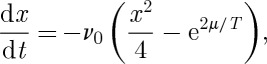
where the first term on the right-hand side accounts for monopole recombination and the second for monopole generation. The recombination rate constant has been assumed to be equal to the monopole hop rate. If the temperature is lowered at a rate d*T*/d*t*=−*r*, then it follows that
11.12
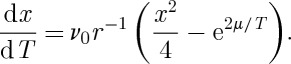
Numerical solution of this equation shows that monopole density reaches a finite approximate steady state of the order *x*=*r*/*ν*_0_ at low temperatures. Putting in reasonable parameters for Dy_2_Ti_2_O_7_ spin ice (e.g. *ν*_0_=1000 s^−1^,*μ*=−4.6 K), a rate of cooling of *r*=10^−5^ K s^−1^ (about 1 K d^−1^) would result in a residual density of about *x*=10^−7^ at temperatures lower that 0.25 K (figure 1). Arrest of the cooling at a base temperature significantly less than 0.25 K, where 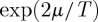 becomes entirely negligible, then results in a very slow power-law decay of the monopole density according to (see equation (11.11))
11.13
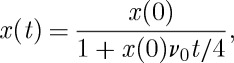
and even 1 day of waiting would barely reduce the density by a further power of 10 (figure 2). Therefore, with any realizable rate of cooling and time of waiting it is not possible to completely rid the system of monopoles on experimental time scales.

The preceding analysis neglects many factors that may become important at low temperatures, including possible thermal evolution of the hop rate, extrinsic factors and kinetic constraints arising from the Dirac strings. However, most of these factors will tend to reduce, rather than increase, the rate of relaxation, so it is safe to conclude that the analysis is correct in its conclusion that a monopole-free state in zero applied magnetic field remains inaccessible to experiment.^6^ A detailed analysis of idealized thermal quenches in spin ice [37] has identified the important role of monopole–antimonopole pairs that cannot immediately annihilate by a single spin flip. These ‘non-contractable’ pairs form long-lived metastable states at low temperature.

It is also pointed out by Castelnovo *et al*. [37] that at sufficiently low temperatures, owing to the divergence of the mobility as 1/*T* (equation (3.2)), monopoles will recombine at the maximum speed allowed by the monopole hop rate. However, in the electrolyte theory, this effect is accounted for by the concept of the Bjerrum pair [11,12]. Such nonlinear response occurs only within the pair, that is, when the monopole–monopole separation is less than *l*_T_=*μ*_0_*Q*^2^/8*πkT*. This fast nonlinear response then appears like the flipping of giant dipoles of magnetic moment *Ql*_T_ [12], but for monopoles at greater separation, the ordinary recombination kinetics of equation (11.13) are obeyed. The average monopole separation grows with decreasing temperature much faster than the Bjerrum pair radius, so, in a ‘slow’ quench of the sort described above, the divergence of the mobility should not significantly speed up the rate of recombination. The role of Bjerrum pairs, which is closely connected to the Wien effect [11,12], is not considered further here.

## 12. Application to experiment: general

In the following sections, I discuss the application of these ideas to different experimental measurements. The equations quoted and derived here should be applicable at sufficiently small applied field and at temperatures (more than 0.4 K for Dy_2_Ti_2_O_7_) where the Wien effect is absent, so the dimensionless monopole density *x* depends only on temperature.

**Figure 3.**
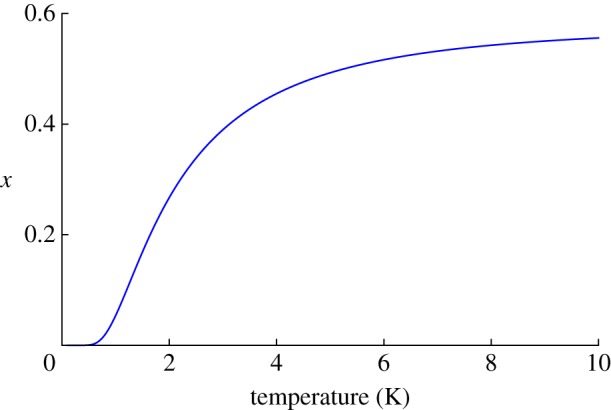
Approximate evolution of the dimensionless monopole density *x* with temperature (J. Bloxsom & S. T. Bramwell 2011, unpublished data) for parameters appropriate to Dy_2_Ti_2_O_7_. The values of *x* have been inferred from fitting experimental specific heat data to Debye–Hückel theory, according to the method of Zhou *et al.* [16].

It is important to emphasize that the experimental response in all cases depends on *x*(*T*)=*ν*/*gν*_0_. There is a general belief that the monopole hop rate *ν*_0_ is temperature independent [7]. Assuming this, *x*(*T*) can be calculated by numerical simulation [7] or by Debye–Hückel theory [17], or approximately inferred from the specific heat [16]. For Dy_2_Ti_2_O_7_, the monopole density is roughly constant below 10 K and decreases rapidly as the temperature is lowered below 2 K (figure 3). The corresponding relaxation time therefore shows a plateau between 10 and 2 K, and increases rapidly as the temperature is further lowered [7]. The picture of monopoles hopping at a constant rate *ν*_0_ breaks down at temperatures above approximately 10 K, where an Orbach-like relaxation process involving an excited crystal field level becomes important [22].

## 13. Magnetization measurements

### 13.1 DC magnetization

To treat a bulk magnetization measurement, we can set *q*=0 in the above equations. In any real sample, demagnetizing fields need to be accounted for. If we assume an ellipsoidal sample and write 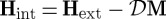 where 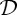 is the demagnetizing factor, then Ryzhkin's equation becomes
13.1
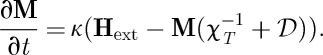
For the case of a steady field this equation may be integrated to find
13.2
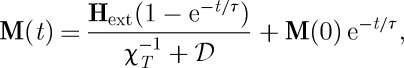
so the relaxation of the magnetization is purely exponential. It may be seen that the susceptibility *χ*_*T*_ behaves as an effective demagnetizing field and that the apparent susceptibility is
13.3
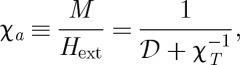
which tends towards 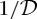 as 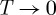.

**Figure 4.**
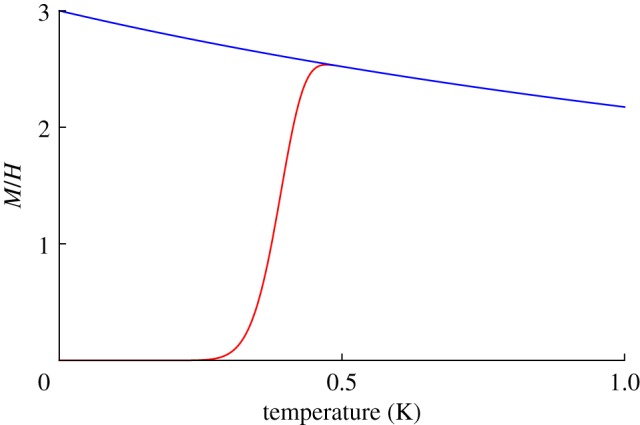
Field cooled (FC, blue) versus zero field cooled (ZFC, red) splitting according to equations (13.4) and (13.5), based on Ryzhkin's theory of monopole current [6]. The observation time has been set at *t*_obs_=100 s, the monopole hop rate at *ν*_0_=10^3^ s^−1^ and the demagnetizing factor at 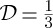. The monopole density has been roughly approximated by *x*≈2 e^*μ*/*T*^/(1+ 2 e^*μ*/*T*^) with *μ*=−4.6 K, appropriate to Dy_2_Ti_2_O_7_.

This equation may be used to describe field cooled (FC) and zero field cooled (ZFC) magnetization measurements. It is assumed that, in the FC experiment, the sample is cooled sufficiently slowly that it always remains in equilibrium (although we have shown that this cannot be strictly true), but that in the ZFC experiment it is heated at a sufficient rate to be observed on a time scale *t*_obs_≪*ν*,*κ*. With these approximations the FC and ZFC magnetizations are
13.4
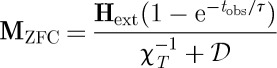
and
13.5
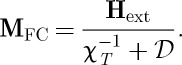
Using reasonable parameters, these equations predict a large FC–ZFC splitting in *M*(*T*)/*H*, as shown in figure 4. Here, it has been assumed that there is a single observation time of about 100 s, which must be a rather crude approximation. Nevertheless, a dramatic FC–ZFC splitting, qualitatively similar to that shown, was observed in experiment by Snyder *et al.* [21]. There appears to be two principal ways in which the experimental result differs from figure 4. First, the experimental FC magnetization below the splitting temperature becomes temperature independent at a value smaller than the theoretical 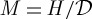 [42]. Second, the experimental splitting temperature (approx. 0.65 K) for Dy_2_Ti_2_O_7_ is higher than that which can be reasonably justified by Ryzhkin's model. The higher than expected splitting temperature appears to be related to an anomalous slowing down of relaxation seen in AC magnetization [19,53], as well as in numerical simulations [7]. Possible causes of the experimentally observed slowing down include the constraints imposed by the Dirac string network [7,37], thermal coupling effects [52] and a transition in the monopole density [54]. Also, the Wien effect is important in this regime and will play a role in the transient response [12,42].

As regards Ryzhkin's prediction [6] that *χ*_*T*_=2*χ*_C_, a recent theoretical study [43], using parameters appropriate to Ho_2_Ti_2_O_7_ spin ice, has shown that there is a very slow cross over between *χ*_*T*_=*χ*_C_ at high temperature (∼100 K) and *χ*_*T*_=2*χ*_C_ in the low-temperature limit. Experimental measurements appear to be consistent with this prediction [43]. This ‘Curie law cross over’ has not yet been experimentally confirmed for Dy_2_Ti_2_O_7_.

### 13.2 AC magnetization

For AC magnetization measurements, Ryzhkin's equation (equation (2.3)) can be applied, using a demagnetization correction. As described above, the rate *ν*=1/*τ*_1_, the spin-lattice relaxation rate that arises in the Bloch equations.

Although Matsuhira *et al.* [20] have shown that the relaxation is never a simple exponential at the temperatures of interest, it appears that the characteristic relaxation time does behave according to Ryzhkin's theory. Thus, at high temperatures we would expect a characteristic relaxation time *τ*=1/*gν*_0_*x* and this is borne out in experiment in the temperature range more than 2 K for Dy_2_Ti_2_O_7_ where 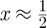 [7]. However, at lower temperatures, it is evident that the relaxation rate may not be simply proportional to the monopole density [7,9,53].

## 14. Neutron scattering

### 14.1 Conventional neutron scattering

Having accounted for the atomic form factor and assuming sufficiently small energy transfer, the partial differential cross section of conventional neutron scattering (*σ*′′) is proportional to the imaginary part of the generalized susceptibility,
14.1
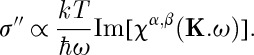
Here, *α*,*β*=*x*,*y*,*z* and ***K*** is the scattering vector. As shown by Fennell *et al*. [13], a polarized neutron scattering experiment may be used to isolate the longitudinal (*zz*) susceptibility discussed here by scanning through a Brillouin zone centre perpendicular to the reciprocal lattice vector ***K***_0_. It is particularly useful to use ***K***_0_=(0,0,2) in the face-centred cubic basis as there is no nuclear Bragg peak at that wavevector [13].

For scans along this direction (which corresponds to a scan across the ‘pinch point’ [13]), using equation (7.4) and setting ***q***=**K**−**K**_0_, we find
14.2
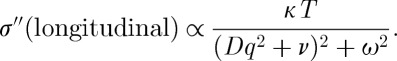
Unfortunately, the dynamics of spin ice are generally too slow to test this expression. Instead, it is possible to energy integrate and measure in the static approximation whereby the differential quasi-elastic cross section *σ*′ is given by
14.3
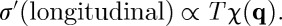


Using equation (7.4) with *ω*=0, we find
14.4
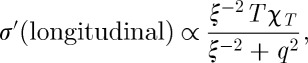
where, as already stated, 
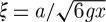
.

In general, *x* is well approximated by
14.5
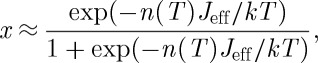
where *J*_eff_ is the effective exchange parameter for a given spin ice [5]. Here, the prefactor 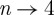 in the low-temperature limit and 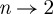 in the high-temperature limit as a result of Debye–Hückel screening [7]. For Ho_2_Ti_2_O_7_ spin ice *J*_eff_/*k*≈1.8 K, so there is a regime at intermediate temperature where 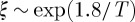. In the experiments of Fennell *et al.* [13], the neutron data along wavevectors perpendicular to 002 were fitted to the sum of a Lorentzian function and a flat background. The inverse Lorentzian width was indeed found to depend on temperature as predicted here (
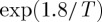
) although its absolute value was much larger than predicted. The flat background was also found to depend on temperature according to equation (14.5) at high temperatures (with *n*=2 and a correction for ‘double charge’ monopoles).

#### 14.1.1 Possible explanation of the discrepancy

There are two potential corrections to equation (14.4) that we did not consider in [13]. The first stems from the modification of equation (7.6) to give equation (7.10), as discussed above. Applying this gives
14.6
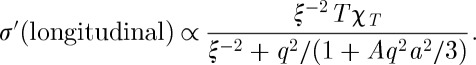
The second would account for the wavevector-dependent misalignment between ***M***(***q***) and ***q***. However, this is a relatively minor correction and is not considered further here. Writing 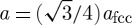, where *a*_fcc_ is the face-centred cubic lattice constant and 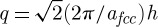, we find (for a scan along *hh*0)
14.7
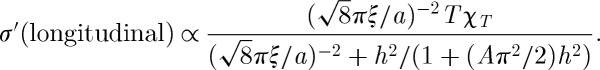
Using equation (7.7), this may also be written
14.8
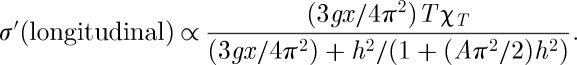
These expressions produce a lineshape and temperature dependence that is very similar to that observed in [13], in that they incorporate both the apparent Lorentzian (making it appear anomalously sharp) and the flat background, and they also predict the correct temperature dependence in both cases. It would be interesting to compare them in detail to the experimental data.

### 14.2 Neutron spin echo

Neutron spin echo measures the intermediate scattering function *S*(***K***,*t*) which is proportional to the frequency Fourier transform of the right-hand side of equation (14.2). Thus, we predict
14.9
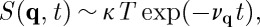
with *ν*_***q***_ given by equation (3.1). A test of this expression would require measuring neutron spin echo for scattering transverse to the pinch point, as above. Experiments so far [22,23] have integrated over larger ranges of ***q***, including transverse fluctuations, and in a temperature range where *ν*_***q***_≈*ν*_0_. A temperature-independent relaxation rate has been observed [22,23], but for Ho_2_Ti_2_O_7_ this was several orders of magnitude faster than that derived by AC susceptibility on Dy_2_Ti_2_O_7_. Notwithstanding a possible variation between materials it seems probable that the measured relaxation rate is technique dependent, even though its temperature dependence is not. This suggests a high-frequency component to the monopole response that is not contained in the present approximations.

## 15. Muon spin relaxation and rotation

### 15.1 Longitudinal field *μ*SR

In a *μ*SR experiment the muon is self-trapped by the lattice distortion it creates. In a dense magnetic oxide such as spin ice it is therefore prone to distort the local magnetic environment that it is aiming to probe. Despite this, the published results of *μ*SR experiments are reasonably explained by the monopole model.

Thus, a longitudinal field *μ*SR experiment on Dy_2_Ti_2_O_7_ was performed by Lago *et al.* [24], who analysed the long-time muon depolarization rate as a measure of the field fluctuation rate. Hence, this should have been a measure of *ν* or 1/*τ*_1_. The temperature dependence of the corresponding relaxation time is indeed very close to that expected, and it seems very probable that the experiment was observing magnetic monopoles. However, the magnitude of the relaxation time was an order of magnitude smaller than that inferred from AC magnetization measurements. This would again suggest a high-frequency component to the monopole response, as noted above.

### 15.2 Transverse field *μ*SR

If a muon implants into spin ice at a site of large local field, then transverse field *μ*SR is an uninteresting probe of the spin-ice system. Hence, we will assume that the muon is at a site of zero local field, either within the sample or exterior to the sample, but near the surface. While the assumption of zero field sites within the spin-ice sample gives a highly consistent description of experiment [11,12], their existence has been contested on theoretical grounds [55] and the issue has been debated [56–58].

At sufficiently high temperature (*T*>10 K), we might expect the transverse field *μ*SR dephasing rate *λ* to give a measure of 1/*τ*_2_, the spin–spin relaxation rate, which may be specified by a BPP-type [59] expression: 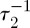,
15.1
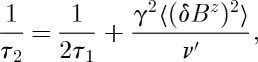
where *ν*′ is approximately the spin flip rate and *δB*^*z*^ is the scale of the fluctuations of the field component parallel to the applied field. As the latter term tends to dominate, we shall only consider this term from now on.

In the spin-ice regime, where *x*=*ν*/*gν*_0_ is the dominating parameter of the system, the transverse field *μ*SR response is found to have a form that is unfamiliar in the context of *μ*SR on paramagnets. To explain this, it is useful to first consider the dimensional analysis of the problem.

### 15.3 Dimensional analysis for transverse field *μ*SR

*μ*SR theory for a simple paramagnet may be formulated in terms of two parameters: Δ and *ν*′. Here, 
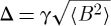
, where the right-hand term is the instantaneous root mean square field at the muon site, *γ* is the muon gyromagnetic ratio and *ν*′ is the relaxation rate of this local field. In terms of dimensional analysis, we would say that *ν*′ and Δ constitute two governing parameters, both with the dimensions of [1/time]. The quantity of interest in transverse field *μ*SR is the characteristic rate of muon dephasing, *λ*. The formal solution to the problem is
15.2
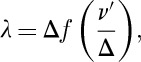
where *f* is an undetermined function.

In the slow fluctuation limit 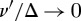 and for *λ* to be finite, we have 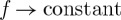. In the fast fluctuation limit, 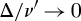 and we expect 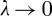. The asymptotic form is in fact linear in the small parameter, *f*(1/*ϵ*)∼*ϵ*. The two solutions thus become
15.3
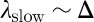
and
15.4
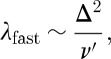
formulae that are often used for the analysis of *μ*SR data.

These formulae may be rationalized by the following heuristic argument. If the field sensed by the muon is approximately static on the muon lifetime, then the muons precess in phase at a Larmor frequency *γB*_*a*_ where *B*_*a*_ is the applied transverse field, but accumulate a phase difference Δ*ϕ*=Δ*t* in time *t*. If 1/*λ* is equated with the time to dephase by order 1 radian, then we obtain *λ*∼Δ. If, on the other hand, the field jumps randomly at rate *ν*, with jump magnitude Δ, then the phase difference accumulated between flips is Δ*ϕ*=Δ/*ν*′ and the phase undergoes a random walk with end-to-end distance *νt*(Δ/*ν*)^2^ in time *t*, yielding equation (15.4).

The case of spin ice is unusual in that there are three, not two, governing parameters. The origin of the third governing parameter is in the thermodynamics of the Coulomb gas in the grand canonical ensemble where the monopole number *N* is the sole extensive system parameter. We have defined *x*=*N*/*N*_0_ as a dimensionless monopole density (where *N*_0_ is the number of diamond lattice sites) and *ν*_0_ is the temperature-independent monopole hop rate. As discussed above, the relaxation rate of the local magnetic field is *ν*=*gxν*_0_ and we may define a scale for the field Δ_0_ that depends only on fixed microscopic parameters.

The formal solution of dimensional analysis can be written
15.5
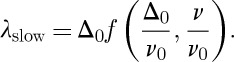
The physical picture we wish to explore is that low temperature (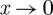) corresponds to slow fluctuations, and high temperature (*x*≈1) corresponds to fast fluctuations. Taking the slow fluctuation limit 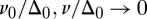 now does not necessarily eliminate *ν*/*ν*_0_=*gx* from the problem. Whether it does so or not depends on the function *f*. If muons detect monopolar fields only (that is, the longitudinal susceptibility), then we would expect *λ* to go to zero as a power law in *x*, for in the absence of monopoles there should be no dephasing. By contrast, in the fast fluctuation limit *ν*/*ν*_0_ does drop out of the problem and we again recover equation (15.4). The two solutions appropriate to the detection of monopolar fields are, therefore,
15.6
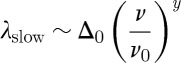
and
15.7
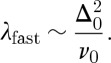


Thus, in the slow fluctuation limit we expect 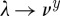, while in the fast fluctuation limit we expect 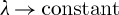. The former is an unusual result in the context of *μ*SR and applies to the case where the muons sense only monopolar fields.

### 15.4 Transverse field *μ*SR at low temperature

At low temperature, the monopole gas is sparse (*x*≪1) and muons that are close to monopoles are rare. The muon experiment acts to some extent as a spectroscopy, associating different field contributions with different times of observation. Hence to use the average field may not be quite correct. The muon signal at long times measures only typical muons, which are far from magnetic monopoles. The typical distance of a muon to a monopole is approximately r*a≈*x*^−1/3^ and the field sensed by the muon is |*B*|≈*B*_0_*x*^2/3^. Since this field is random in direction, we get the same result for the mean square field as above, but with the exponent 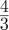 on *x* instead of 1. In general, we might expect the apparent mean square field to be given by the equation 
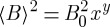
, with *y*≈1.

In this limit, the Debye length *l*_D_ is very large and the conductivity *κ* is very small. Although *κ* scales with *x*, if *y*<2, then 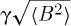 is always larger than it, and the fields are quasistatic (here *γ* is the muon gyromagnetic ratio). If we approximate the fields as completely static on the muon lifetime, then the muons sense a *z*-component of the local field that is of the order of the root mean square field. The field sensed by the muons is approximately 
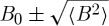
 and the muons precess coherently at a Larmor frequency *γB*_0_ but are dephased by the spread in local fields. Introducing 
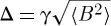
 the spread of phases accumulated in time *t* is
15.8
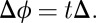
The dephasing time 1/*λ* is equated with the time taken for Δ*ϕ* to become of order one radian, with the result^7^
15.9
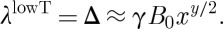
Hence, *λ* is
15.10
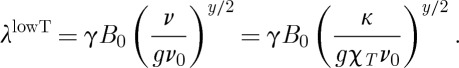


The muon dephasing function depends on the actual field distribution. However, it is always of the form
15.11
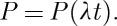
This form (with *λ*∝*ν*) was assumed in Bramwell *et al*. [11] and gave a highly consistent description of experiment. Although this applies the current ideas in the Wien effect regime, one would expect this to be reasonable on the grounds of the dimensional arguments given above. Note also that the method of Bramwell *et al*. [11] is insensitive to the precise form of the local field distribution. The typical value of *λ* observed in [11] was of the order 10^5^ s^−1^. For Dy_2_Ti_2_O_7_ spin ice *γB*_0_≈10^8^ s^−1^, so a *λ* of 10^5^ s^−1^ corresponds to *x*=10^−6^ if *y*=1 and the monopole field at a typical muon site is about 10^−3^ T. The temperature at which the monopole density is expected to fall to this value is 0.3 K, which is consistent with the observations of Bramwell *et al.* [11].

### 15.5 Transverse field *μ*SR at high temperature

In the high-temperature limit *x* becomes of order unity so *ν*≈*ν*_0_. Thus, as we pass from low to high temperature, monopoles hopping at a rate *ν*_0_ located near the muon become increasingly important, but, as remarked above, these monopoles cannot be distinguished from spins, and we return to a model of spin flipping at rate *ν*_0_. In this case, the ordinary equations of *μ*SR apply.

## 16. Conclusion

The main conclusion of the present work is that magnetic monopoles in spin ice largely determine the longitudinal response of the system. The sole system variable for both static and dynamic response is the dimensionless monopole density *x*, which is determined in a complex way by the four fixed parameters of the problem: *a*,*Q*,*μ* and *ν*_0_. In contrast, the transverse response does not directly mirror monopole correlations.

The main theoretical results of this paper are contained in equations (3.6), (5.8), (6.3), (7.5), (7.7), (8.4), (10.1), (9.2) and (9.3).

Temporal and spatial correlations are linked by *x* and equations (3.6) and (7.7) combine to establish a dynamic scaling relation
16.1
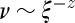
with *z*=2, as would be expected for a problem of Brownian motion. It follows (see equation (7.5)) that there is a dispersion of relaxation rates on all scales from the monopole hop rate *ν*_0_ to the magnetization relaxation rate *ν*. Some evidence has been noted to suggest that field fluctuations relax more quickly than spin fluctuations (see equations (7.5) and (10.1)) but more work is needed to establish this.

The exponents *ν* and *z* defined in this way, and the correlation length *ξ*, are not conventional quantities as they reflect monopole rather than spin correlations. The spin correlations obey static scaling in the following sense. The correlation function *g*(*r*), being pseudo-dipolar [36,44], decays as *g*(*r*)∼*r*^−3^. Applying the scaling relation *g*(*r*) *r*^−(*d*−2+*η*)^ we find *η*=2. As the susceptibility diverges as 1/*T*, the susceptibility exponent *γ* takes the value *γ*=1. Applying the scaling relation *ν*=*γ*/(2−*η*), we find *ν* is infinite, meaning that the spin–spin correlation length remains finite at all temperatures. Thus, *T*=0 marks an unusual critical point with algebraic decay of spin correlations, a divergent spin susceptibility, but a non-divergent spin correlation length. It is interesting to observe, however, that the monopole correlation length does diverge at *T*=0.

Free energy functionals for the magnetization and field fluctuations have been derived (equations (6.3) and (8.4)) and shown to relate closely to equation (5.8), previously stated by Ryzhkin and Ryzhkin [38]. In future work, it would be interesting to express these as functionals of the density (*x*) and to add further terms to account for energy fluxes in the system, as well as Wien dissociation, which both play a role at low temperature [11,12,18].

The generalized susceptibility (equation (7.5)) at the level of Ryzhkin's description [6] has been derived, as well as the field fluctuation at the level of Debye–Hückel theory [17]. The latter was used to calculate the longitudinal field fluctuation at a point in the system (equations (9.2) and (9.3)), which may be compared with established results for electrolytes [49]. The expressions for the mean square field distribution have been used to show that a point probe such as a muon at a ‘spin free’ site (either inside the sample or just outside) will give a direct measure of the monopole density as assumed in Bramwell *et al*. [11].

It has been shown that, according to the electrolyte theory, a non-equilibrium population of monopoles is always frozen into the sample, regardless of the rate of cooling. However, in sufficiently weak magnetic field there is never a phonon bottleneck. The former effect should generally be considered when treating low-temperature experimental data.

In general, the theory discussed here works qualitatively well for real spin-ice materials, capturing the temperature, wavevector and time dependence of a diverse range of experimental responses. However, there are three clear discrepancies. First, while the temperature and wavevector dependence of the neutron scattering cross section are well accounted for, the amplitude of the correlation length is not, being an order of magnitude longer in experiment than in theory [13]; a possible explanation of this has been proposed here. Second, while different experiments [20–24] agree on the temperature dependence of the relaxation rate, they exhibit a wide range of relaxation rates: it appears that there is a high-frequency response, not accounted for in the hopping model. Third, the AC susceptibility relaxation in the high-temperature limit is more strongly dispersed [20] than predicted by the simple approximations discussed here.

It seems very unlikely that the monopole theory will have to be abandoned to explain these discrepancies. More probably, it needs to be refined. In addition to the possible revision of the neutron scattering line shape discussed above, one might also need to consider the effect of quantum fluctuations [47,48] or minor terms in the spin-ice Hamiltonian [32]. Also, microscopic factors affecting the rate of local spin flipping or monopole hopping probably remain to be identified. However, it should also be emphasized that the most distinctive aspect of Coulombic correlation—the tendency to Bjerrum pairing—has not been accounted for here, or in other ‘high-temperature’ theories, but will certainly play a role. Thus, Bjerrum pairs have been argued to be important in the low-temperature non-equilibrium regime [12] and have been identified in specific heat measurements [16]. Finally, the Wien effect [60], though weak at the ‘high’ temperatures considered here, still exists in a screened form [61], and should be accounted for in a more accurate description. Although there is much work to be done, it is clear that the monopole theory of spin ice [1,6] is a remarkably simple and effective description of a complex condensed matter system.

## Footnotes

1 In spin ice, the DC Wien effect takes the form of a transient increase in charge density under applied field, before equilibration to a value lower than the zero field value (V. Kaiser & P. C. W. Holdsworth 2010, personal communication); the AC Wien effect should take the form of a steady-state increase in current.

2 A detailed discussion of the dynamical susceptibility of spin ice in the vicinity of the critical point induced by a field of *μ*_0_*H*≈1 T applied along the [111]-axis [39] has been given by Shtyk & Feigel’man [40].

3 The Nernst–Einstein equation, which may be derived from the Boltzmann transport equation, is one of the basic equations of electrochemistry. According to Wannier [41], it was used by Nernst in 1888 to make the first direct measurement of the elementary electronic charge—at a time when the electron had not yet been identified and even the existence of atoms or ions was controversial. The Nernst–Einstein equation may be used in this way because diffusivity and mobility are independently measurable for an electrolyte. However, for magnetic monopoles in spin ice no way has yet been identified to measure these quantities independently.

4 In the temperature range considered here (e.g. 0.4–10.0 K for Dy_2_Ti_2_O_7_), at zero applied field, the Debye length is the dominant length scale in the system, and, as shown below, magnetic inhomogeneities develop on this length scale. In the lower temperature range, not considered here, inhomogeneities on the scale of the Bjerrum length *l*_T_=*μ*_0_*Q*^2^/8*πkT* become particularly important, and probably dominate (see [11,12,42]).

5 The functional derivative of  is found as follows:

6.5

where the angular brackets indicate a distribution with vector test function ***f***(***r***). Now

6.6

so, using equation (5.3) and the divergence theorem,

6.7

where *S* denotes a surface integral, ***n*** a unit normal to the surface and *a* a surface element. If the surface charge is everywhere zero then we have

6.8

and finally

6.9

6 One may contrast the case of pure water at room temperature, where a density of ∼10^−9^ H^+^ ions per water molecule is easily maintained at equiliibrium on experimental time scales; however, the diffusion constant of H^+^ in water is some 10^8^ times larger than that of magnetic monopoles in spin ice.

7 Henley (C. Henley 2011, personal communication) has considered relaxation functions *G*(*t*) for NMR for nuclei in sites of zero local field, and in the dilute monopole limit has shown that  where *β* is a positive exponent of order unity and *τ*_*i*_ (*i*=1,2) is a characteristic time scale for longitudinal (1) and transverse (2) relaxation. The results obtained here (for zero local field sites) are consistent with the *μ*SR relaxation function taking this general form.
